# Clinical Diagnosis and Geographic Distribution of Leptospirosis, Thailand

**DOI:** 10.3201/eid1301.060718

**Published:** 2007-01

**Authors:** Vanaporn Wuthiekanun, Nisa Sirisukkarn, Prayad Daengsupa, Prangyong Sakaraserane, Amornwadee Sangkakam, Wirongrong Chierakul, Lee D. Smythe, Meegan L. Symonds, Michael F. Dohnt, Andrew T. Slack, Nicholas P. Day, Sharon J. Peacock

**Affiliations:** * Mahidol University, Bangkok, Thailand; † Ministry of Public Health, Thailand; ‡ Queensland Health Scientific Services, Brisbane, Australia; § University of Oxford, Oxford, United Kingdom

**Keywords:** Leptospirosis, clinical diagnosis, positive predictive accuracy, geographic distribution, Thailand, dispatch

## Abstract

We defined the positive predictive accuracy of a hospital-based clinical diagnosis of leptospirosis in 9 provinces across Thailand. Of 700 suspected cases, 143 (20%) were confirmed by laboratory testing. Accuracy of clinical diagnosis varied from 0% to 50% between the provinces and was highest during the rainy season. Most confirmed cases occurred in the north and northeast regions of the country.

Leptospirosis is an emerging infectious disease in Thailand (*1*). Before 1996, the number of cases reported to the Thailand Department of Disease Control (DDC) was ≈200 per year. Leptospirosis was sporadic and reported mainly in central and southern regions. A marked change occurred in the decade thereafter, with an increase from 358 cases in 1996 to a peak of 14,285 cases in 2000. This was followed by a continual decline to 2,868 cases in 2005 (*1*). Most cases (90%) throughout this period were reported in northeast Thailand. A study of >600 adults who sought treatment for fever at 1 hospital on the Thai-Myanmar border provided further evidence of the importance of leptospires as an important pathogen in this region, with serologic evidence for leptospirosis found in 17% of the patients (*2*). The true extent of the disease is likely considerable in Thailand, which illustrates the need for accurate epidemiologic tools for its evaluation. An essential part of this process is understanding the mechanisms of reporting and their inherent inaccuracies.

Reporting of leptospirosis to the DDC in Thailand is voluntary. During a review of the national surveillance system for leptospirosis in 2 northeastern provinces, interviewed physicians said the national case definition was difficult to understand and apply (*3*). Investigators concluded that the lack of a standardized case definition for leptospirosis; the infrequent use of confirmatory laboratory testing; and the inability to link clinical, epidemiologic, and laboratory data hindered the system’s utility (*3*). These results imply that both underreporting and diagnostic inaccuracy of reported cases may be occurring. We conducted a prospective multicenter study to define the accuracy of clinical diagnoses of suspected leptospirosis in Thailand and to describe the geographic distribution of laboratory-confirmed cases.

## The Study

From March 2003 though November 2004, admitting physicians in district and provincial hospitals within 9 provinces of Thailand in the north, northeast, central, and southern regions were invited to recruit patients of all ages suspected on clinical grounds to have leptospirosis. Clinical features considered were those specifically referred to in the national guidelines (e.g., fever, headache, muscle pain, meningism, conjunctival suffusion, and jaundice) together with hemoptysis, hepatomegaly, diarrhea, hypotension, and reduced urine output. From each patient, a 5-mL serum sample was taken to be cultured for *Leptospira,* another 5-mL serum sample was taken for serologic testing, and a third sample was taken 2 weeks later for serologic testing. Serum was stored at –80°C until analysis.

Microscopic agglutination test (MAT) was performed at the World Health Organization (WHO)/United Nations Food and Agriculture Organization (FAO)/World Animal Health Organisation (OIE) Collaborating Center for Reference and Research on Leptospirosis, Brisbane, Queensland, Australia (*4*). A positive MAT was defined as a single titer of >1:400 or a 4-fold rise in titer between acute and convalescent phase samples. For *Leptospira* culture, 100 μL of whole blood, 500 μL of plasma, and 500 μL of serum were each injected into 3 mL of Ellinghausen, McCullough, Johnson, and Harris (EMJH) medium and supplemented with 3% rabbit serum and 0.1% agarose, then incubated aerobically at room temperature (25°C–30°C) for 6 months and examined every week for 2 months, every 2 weeks during months 3 and 4, and once a month during months 5 and 6. Examination was done by placing 1 drop of culture onto a microscopic glass slide and viewing by dark-field microscopy at 200× magnification. Positive cultures were referred to the WHO/FAO/OIE Collaborating Center for Reference and Research on Leptospirosis for identification by using the cross-agglutination absorption test (*4*).

A total of 700 patients with a clinical diagnosis of leptospirosis were recruited during the study period. All patients had blood samples collected at the hospital for leptospire culture and serologic testing; convalescent-phase serum samples were obtained during follow-up for 509 (73%) patients.

**Figure 1:**
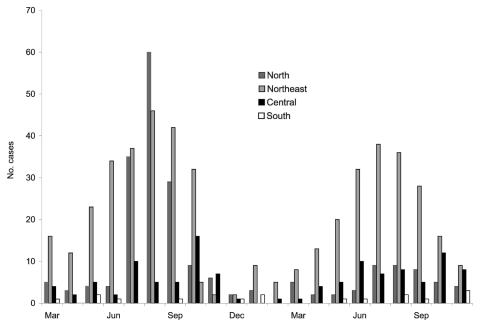
Cases of clinical leptospirosis by month for each geographic region, Thailand, March 2003–November 2004.

The median age of patients with suspected leptospirosis was 38 years (range 2–95 years, interquartile range (IQR) 28–49 years); 504 (72%) were men. The number of clinically diagnosed leptospirosis cases by month in the north, northeast, central, and southern regions is shown in Figure 1. Most cases (597, 85%) were recorded in 4 provinces in the north or northeast (Table). Cases were predominantly identified during the rainy season (June–October) in the north and northeast in 2003, with a second peak in the northeast, but not the north, during the rainy season of 2004. Little variation occurred over time in the central and southern regions.

**Table:** Distribution of suspected and confirmed cases of leptospirosis according to province, Thailand

Province	Geographic region	Clinically suspected cases (%)*	Laboratory- confirmed cases (%)†	Positive predictive accuracy (95% CI)‡	Culture-positive cases
Lumpang	North	161 (23)	28 (20)	17% (12–24)	2
Udon Thani	Northeast	223 (32)	64 (45)	29% (23–35)	10
Maha Sarakham	Northeast	181 (26)	26 (18)	14% (10–20)	1
Ya Sothon	Northeast	32 (5)	6 (4)	19% (7–36)	1
Chainut	Central	13 (2)	3 (2)	23% (5–54)	0
Rayong	Central	45 (6)	13 (9)	29% (16–44)	1
Chanthaburi	Central	4 (0.6)	2 (1)	50% (7–93)	0
Prachuap Khiri Khun	South	33 (5)	1 (0.7)	3% (0.1–16)	0
Phattalung	South	8 (1)	0	0% (0–37)	0
Total		700	143	20%	15

*Percentage of total suspected cases. †Percentage of total confirmed cases. ‡CI, confidence interval.

Of the 700 patients who received a clinical diagnosis of leptospirosis, 143 (20%) received a confirmed diagnosis of leptospirosis based on *Leptospira* isolation, MAT testing, or both (Table). The median age of patients with confirmed leptospirosis was 35 years (range 10–68 years, IQR 27–45 years); 121 (85%) were men. The diagnosis was confirmed after isolation of leptospires from 15 (11%) patients; the geographic distribution is shown in the Table. The serovars of cultured *Leptospira* were: *L. interrogans* serovar (sv.) Autumnalis (7), *L. interrogans* sv. Bataviae (2), *L. interrogans* sv. Pyrogenes (2), *L. borgpetersenii* sv. Javanica (1), *L. interrogans* sv. Hebdomadis (1), *L. interrogans* sv Grippotyphosa (1), and an unidentified serovar (1). An additional 128 patients with culture-negative samples had been exposed to *Leptospira* as determined by MAT; results for 96 (75%) were based on a 4-fold rising titer and for 32 (25%), on a single raised titer of >1:400.

**Figure 2:**
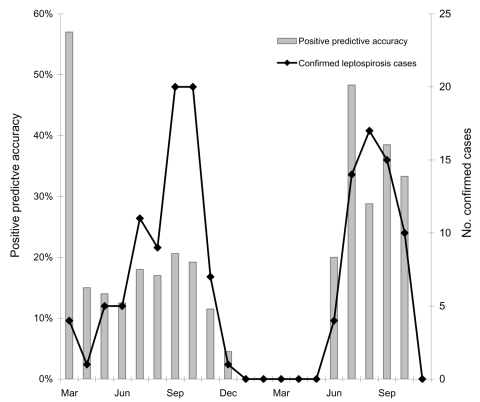
Cases of laboratory confirmed leptospirosis and positive predictive accuracy of clinical diagnosis by month, Thailand, March 2003–November 2004.

The geographic distribution of the 143 laboratory-confirmed cases is summarized in the Table. Most of these patients (124, 87%) lived in the 4 provinces found in the north and the northeast (Table). The month of diagnosis for confirmed cases is shown in Figure 2; most were during the rainy season.

The positive predictive accuracy of a clinical diagnosis is defined by the number of laboratory-confirmed cases divided by the number of clinically suspected cases. Results for each of the 9 provinces are shown in the Table. When only data from centers that reported at least 10 cases were used, positive predictive accuracy ranged from 3% to 29%. Positive predictive accuracy by month of study is shown in Figure 2.

## Conclusions

Diagnosing leptospirosis at the point of care is notoriously difficult in the tropical setting, where several common infectious diseases are often hard to differentiate. Positive predictive accuracy for leptospirosis was highest during the rainy season, an observation that is likely related to the higher disease incidence and pretest probability. Variability in positive predictive accuracy was seen among the 3 provinces with the highest number of both suspected and true cases. The reason for this is unclear but may relate to perceived risk to the community, local policy, or other factors.

The finding that both clinical and confirmed cases of leptospirosis were more common in the north and northeast is consistent with DDC reports. Increased incidence in this region may have resulted from >1 events, such as an increase in the rodent population, a natural reservoir for this pathogen, and a population in which around one third are positive for *Leptospira* in northeast Thailand (*5*). Alternatively, 1 clone or a small number of bacterial clones may have become adapted for persistence at greater numbers within the natural host or in the environment. These factors could increase the leptospire count in contaminated water. It is also possible that 1 clone or a small number of clones have become adapted for enhanced invasion of the human host. The most prevalent serovar isolated was *L. interrogans* serovar Autumnalis (7/15 [47%] isolates), 6 of which were from cases in the north or northeast. Further genomic analysis is required to determine whether clonality exists among these isolates.

The effect of the low level of accuracy of hospital-based clinical diagnosis of leptospirosis in rural Thailand is not known. A common disease in this setting that is easily confused with leptospirosis is scrub typhus; both diseases would be predicted to respond to doxycycline, an antimicrobial drug often prescribed for undifferentiated fever. Further studies are required to define the implications of our findings and determine whether routine laboratory testing for leptospirosis should be implemented in Thailand.
